# Unexpected Clinical and Laboratory Observations During and After 42-Day Versus 84-Day Treatment with Oral GS-441524 in Cats with Feline Infectious Peritonitis with Effusion

**DOI:** 10.3390/v17091181

**Published:** 2025-08-29

**Authors:** Katharina Buchta, Anna-Maria Zuzzi-Krebitz, Michèle Bergmann, Roswitha Dorsch, Katharina Zwicklbauer, Kaspar Matiasek, Regina Hofmann-Lehmann, Marina L. Meli, Andrea M. Spiri, Yury Zablotski, Martin Alberer, Ulrich von Both, Katrin Hartmann

**Affiliations:** 1 LMU Small Animal Clinic, Centre for Clinical Veterinary Medicine, LMU Munich, 80539 Munich, Germany; fipmunich@gmail.com (A.-M.Z.-K.); michele.bergmann@lmu.de (M.B.); roswitha.dorsch@lmu.de (R.D.); k.zwicklbauer@medizinische-kleintierklinik.de (K.Z.); y.zablotski@med.vetmed.uni-muenchen.de (Y.Z.); hartmann@lmu.de (K.H.); 2 Institute of Veterinary Pathology, Centre for Clinical Veterinary Medicine, LMU Munich, 80539 Munich, Germany; kaspar.matiasek@neuropathologie.de; 3 Clinical Laboratory, Department of Clinical Diagnostics and Services, Center for Clinical Studies, Vetsuisse Faculty, University of Zurich, CH-8057 Zurich, Switzerland; regina.hofmann-lehmann@uzh.ch (R.H.-L.); mmeli@vetclinics.uzh.ch (M.L.M.); aspiri@vetclinics.uzh.ch (A.M.S.); 4 Division of Paediatric Infectious Diseases, Dr. von Hauner Children’s Hospital, University Hospital, LMU Munich, 80337 Munich, Germany; martin.alberer@lrz.uni-muenchen.de (M.A.); ulrich.von.both@med.uni-muenchen.de (U.v.B.); 5 German Center for Infection Research (DZIF), Partner Site Munich, 80337 Munich, Germany

**Keywords:** feline coronavirus, cats, FCoV, FIP, therapy, antiviral chemotherapy, side effects

## Abstract

The nucleoside analogue GS-441524 is a common treatment for cats with feline infectious peritonitis (FIP). In a previous study, 40 cats with FIP with effusion were treated with 15 mg/kg GS-441524 orally once daily for either 42 days or 84 days, and a 42-day treatment was as effective as the earlier recommended 84-day treatment. The aim of the present study was to describe unexpected clinical and laboratory observations occurring during and after treatment (within one year) in these cats and to compare them regarding the different treatment durations. Thirty-eight cats recovered rapidly during treatment, two cats had to be euthanized, and one cat was lost to follow-up. During treatment, 25 cats developed diarrhea. Lymphocytosis occurred in 26/40 cats during treatment, eosinophilia in 25/40 during treatment, increased alanine aminotransferase activity in 22/40, alkaline phosphatase activity in 7/40, and symmetric dimethylarginine levels in 25/40. These unexpected observations occurred equally in both treatment duration groups, but statistically significantly more cats developed lymphocytosis and eosinophilia when treated for 84 days. Although most of the unexpected observations during GS-441524 treatment improved or disappeared after treatment termination, these conditions have to be monitored, and treatment should not be given for longer than necessary.

## 1. Introduction

Feline infectious peritonitis (FIP) was a highly fatal disease [1] until recently, as antiviral drugs such as the nucleoside analogue GS-441524 now offer a chance of cure [2]. GS-441524, the active metabolite of remdesivir [3], is an antiviral compound with high efficacy against FIP, as already demonstrated in several studies of the authors’ research group [4,5,6,7], as well as by others [2,8,9,10,11,12,13,14,15,16,17,18,19]. Within a short period of antiviral treatment, cats showed rapid improvement in both clinical and laboratory parameters, along with a fast decrease in viral loads. A prospective, randomized controlled trial conducted by the authors’ research group demonstrated that a 42-day (six-week) course of oral GS-441524 is sufficient to induce complete remission in cats affected by FIP. Cats were randomly divided into two groups, with 20 cats receiving treatment for 42 days and the other 20 cats for 84 days; 38/40 cats were in complete remission for up to 168 days (24 weeks) [6].

During treatment with orally administered GS-441524, unexpected observations, such as lymphocytosis, eosinophilia [4,17,18], elevation in liver enzyme activity, e.g., alanine aminotransferase (ALT) and alkaline phosphatase (ALP) [4,17], and/or an increase in symmetric dimethylarginine (SDMA) [2,4,9] have been reported in some cats. Two cats that developed urolithiasis during treatment with illegally produced GS-441524 have been described [20]. Thus, monitoring unexpected observations during treatment is essential.

As unexpected observations can occur during and after the antiviral treatment, it is important to evaluate whether a shorter treatment duration is associated with a lower risk of such findings. Therefore, the aim of the present study was to describe unexpected clinical and laboratory observations occurring during and up to one year after the treatment start with GS-441524 and to compare these observations between the long and short treatment duration groups of cats described in the study of Zuzzi-Krebitz and colleagues (2024) [6].

## 2. Materials and Methods

### 2.1. Study Design and Cohort

This study followed up on a previously published study by Zuzzi-Krebitz and colleagues (2024): A total of 40 cats with FIP were originally included in the study [6] (Table 1) based on the following criteria: (1) presence of effusion; (2) diagnosis of FIP (positive reverse transcription quantitative PCR (RT-qPCR) from effusion in combination with clinicopathological abnormalities typical for FIP); (3) body weight of at least two kg; (4) negative feline leukemia virus (FeLV) antigen and feline immunodeficiency virus (FIV) antibody test; and (5) absence of other severe diseases independent of FIP [6]. All 40 cats were treated with GS-441524 (provided by BOVA Specials, London, UK) at the same dosage of 15 mg/kg per os (PO) every 24 h (q24h). Cats were randomly assigned to two groups: 20 cats received GS-441524 for 42 days (short treatment group), while the other 20 cats were treated with GS-441524 for the currently recommended longer treatment duration of 84 days (long treatment group), as already described in the first publication on this cohort of cats [6].

The cats stayed in the hospital from day 1 (treatment start) to day 7, with daily monitoring of activity, breathing rate, food/water intake, fecal/urine excretion, and body weight, and were discharged on day 7 after the treatment start if clinically appropriate. Cats were frequently examined until day 84 (days 14, 28, 42, 56, and 84) and followed up for one year with rechecks on days 168 (24 weeks), 252 (36 weeks), and 365 (52 weeks) after the treatment start. Overall, 38 cats completed the treatment. Two cats had died during treatment (days 3 and 31) and underwent full postmortem examination, including necropsy, histological workup, and immunohistochemical examination for feline coronavirus (FCoV) antigen and FCoV RT-qPCR [6]. One cat was lost to follow-up due to lack of owner compliance. Thus, unexpected clinical and laboratory observations could be prospectively evaluated in a total of 37 cats in this study.

### 2.2. Monitoring of Cats

**Figure 1:**
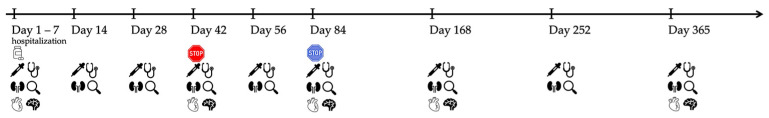
Timeline visualizing the examinations during the hospitalization (day 1–7) and follow-up throughout the study course. 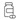: start of treatment with GS-441524 (day 1), 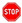: end of treatment of 20/40 cats on day 42, 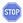: end of treatment of 20/40 cats on day 84, 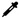: blood tests (including hematology, serum biochemistry, and SDMA), 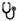: physical examination, 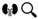: abdominal ultrasonography, 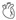: detailed cardiologic examination (including electrocardiography and echocardiography), 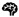: neurologic examination.

Before the treatment start with GS-441524 (day 1), all cats underwent a thorough evaluation, including a detailed medical history, physical examination including the modified Karnofsky’s score [21], and abdominal ultrasonography, as well as detailed cardiologic and neurologic examination. If cystocentesis was possible, urinalysis was performed (not in cats with thrombocytopenia or small bladder size). Hematological and clinical chemistry laboratory analytes, including SDMA, were measured. Throughout hospitalization (days 1–7) and on days 14, 28, 42, 56, 84, 168, 252, and 365, physical examinations were performed, and laboratory analytes were monitored (Figure 1). Abdominal ultrasonography was performed using the Logiq E9 ultrasound system (GE Healthcare) with an 8-MHz microconvex and 5–18 MHz hockey-stick probe. The procedure was performed with the cat in dorsal recumbency after fur clipping. On day 7, most cats were discharged from the hospital; twelve cats stayed in the hospital a few days longer due to diarrhea or laboratory changes that required treatment or monitoring. Owners were required to maintain a diary during the first 84 days after the treatment start to record activity, breathing rate, food/water intake, fecal/urine excretion, and body weight daily. Fecal consistency was evaluated daily using the Purina fecal score, Nestlé Purina, St. Lous, MO, USA [22]. To exclude parasitic infections, fecal samples were analyzed using the Vetscan Imagyst^®^ (Zoetis, Berlin, Germany) FEC OVA/OCC Kit (for detection of worm eggs and coccidia; glucose solution) and the Vetscan Imagyst^®^ FEC GIARDIA Kit (for detection of Giardia species; zinc sulfate solution) with the Vetscan Imagyst^®^ system (Zoetis), according to the manufacturer’s instructions.

Hematological blood analyses were performed using automated analyzers (Cell-Dyn 3500, Abbott Laboratories, Chicago, IL, USA, and ProCyte Dx, IDEXX Laboratories Inc., Westbrook, ME, USA). A manual differential blood cell count was conducted on blood smears stained with Haema Quik/Diff-Quik. Serum biochemical analytes were measured using an automated analyzer (Hitachi 911, Roche, Grenzach-Wyhlen, Germany). Serum creatinine levels were interpreted based on the IRIS guidelines [23]. SDMA was quantified at IDEXX Diavet AG (Bäch, Switzerland) utilizing a high-throughput immunoassay, and SDMA levels were interpreted in accordance with IDEXX^®^ recommendations, defining values above 14 µg/dL as elevated.

Urinalysis was conducted with the use of automated analyzers (VetLab UA Analyzer and SediVue Dx Urine Sediment Analyzer, IDEXX Laboratories Inc., Westbrook, ME, USA). The urine specific gravity (USG) was assessed using a feline-calibrated refractometer. The urine protein creatinine (UPC) ratio was measured using an automated analyzer (Hitachi 911, Roche, Grenzach-Wyhlen, Germany).

### 2.3. Unexpected Clinical and Laboratory Observations

Unexpected clinical and laboratory observations were defined as any abnormalities regarding clinical or laboratory parameters during and after treatment with GS-441524. In this study, the term “unexpected observations” refers to any undesirable or unexpected clinical or laboratory finding occurring during or after antiviral treatment. These observations could have been caused by the antiviral treatment, but establishing a definitive causal relationship can be difficult.

These unexpected observations were classified into two groups: “during treatment” and “after treatment”. Given the two different treatment durations, “during treatment” referred to cats in the short treatment group up to day 42, while “after treatment” was applied to the follow-up assessments on days 56, 84, 168, 252, and 365, respectively (Table 2). For cats in the long treatment group, “during treatment” included evaluations up to day 84, with “after treatment” referring to assessments on days 168, 252, and 365 (Table 3).

### 2.4. Statistical Analysis

Due to repeated measurements for an individual animal on multiple days, most variables were analyzed using linear mixed-effects models, with an individual animal as a random effect. The eosinophil count and the Purina fecal score were analyzed using median mixed-effects models. The following model assumptions were assessed: (1) normality of residuals was evaluated using the Shapiro–Wilk normality test, (2) homogeneity of variances between groups was tested with Levene’s test, and (3) heteroscedasticity (constancy of error variance) was checked with the Breusch–Pagan test. If any of these assumptions were violated, a robust mixed-effects linear model (RLMER) was applied. RLMER computes weighted estimates via Design Adaptive Scale and thus solves heteroskedastic and non-normally distributed residuals by assigning lower weights to outliers and other contaminations (outliers and influential points). Model fit was assessed for both standard and robust models using coefficients of determination (R^2^), intraclass correlation coefficient (ICC), and root mean squared error (RMSE), with preference given to models demonstrating the best fit. ALT levels and the Purina fecal score were log-transformed to correct for strong skewness in their distributions. The eosinophil count and the Purina fecal score were analyzed using median mixed-effects models due to a skewed distribution, low numbers, and non-parametric (score) nature. All contrasts (differences) between particular days and duration categories were assessed after model fitting using estimated least-squares marginal means (emmeans) with the Bonferroni *p*-value correction for multiple comparisons. Results were considered statistically significant at *p*-value < 0.05 and suggestive at *p*-value < 0.1. All models were conducted and figures generated using R statistical language (version 4.3.1 (16 June 2023)).

Heatmaps were generated to visualize Purina fecal score, eosinophil count, lymphocyte count, liver enzyme activity, and SDMA levels of individual cats over time using Microsoft^®^ Excel version 16.92 (24120731).

## 3. Results

During treatment with GS-441524, one or more unexpected observations were detected in all cats at different time points (Table 4). In some cats, unexpected observations were found both during and after treatment.

### 3.1. Diarrhea

**Figure 2:**
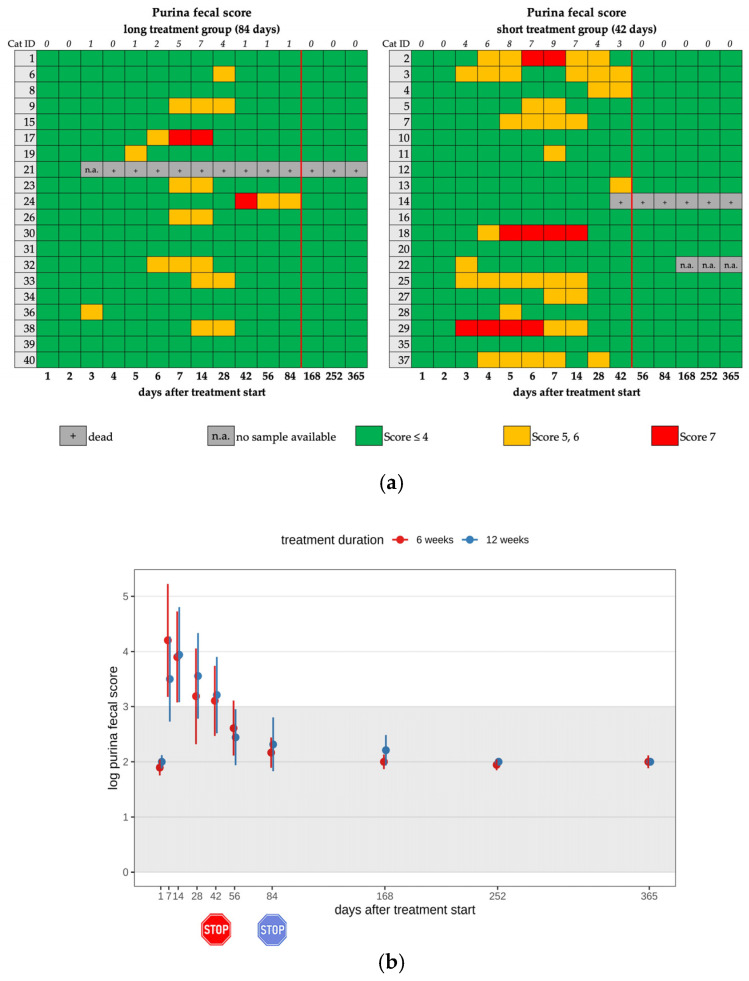
(**a**) Heatmap visualizing the Purina fecal score in cats throughout the study period in both groups until day 365 (comparison of treatment duration: 42 versus 84 days). A score of ≤4 (green) indicates normal fecal consistency, and a score of 5 or 6 (yellow) indicates mild diarrhea. A score of 7 (red) was defined as severe diarrhea. The red line indicates the end of the treatment period, and the line above the map represents the number of cats with a Purina fecal score equivalent to diarrhea. (**b**) Timeline visualizing changes in the Purina fecal score of the cats during the treatment with GS-441524 until day 42 (red) or day 84 (blue) and up to day 365 (comparison of treatment duration: 42 versus 84 days). The red stop sign marks the end of treatment of the short treatment group; the blue stop sign marks the end of treatment of the long treatment group. Figures show average predictive values and 95% confidence intervals of each parameter. Grey shading marks the reference interval of the parameters. There was no statistically significant difference in group comparison measured by a median mixed-effects model. The Purina fecal score was log-transformed to correct for strong skewness in its distribution.

Fourteen cats (14/25) in the short and eleven cats (11/25) in the long treatment group developed diarrhea during treatment with GS-441524 (25/40; 62.5%). Diarrhea only occurred during (and not after) treatment and in both groups (Table 2, Table 3 and Table 4). There was no significant difference observed between the two groups. In five cats, diarrhea was severe, corresponding to a Purina fecal score of seven (Nestlé Purina, St. Louis, MO, USA) (Figure 2a,b) [22]. Fecal examinations were performed in all cats with diarrhea to exclude parasite infestation; all 25 cats tested negative. In 8/25 cats, diarrhea occurred for only one day during the treatment. Cats with severe diarrhea received probiotics and fluid therapy. However, during the follow-up examinations after treatment discontinuation, no cat had diarrhea. Evaluation of FCoV shedding in feces for the long-term follow-up was not performed in the present study.

### 3.2. Lymphocytosis

**Figure 3:**
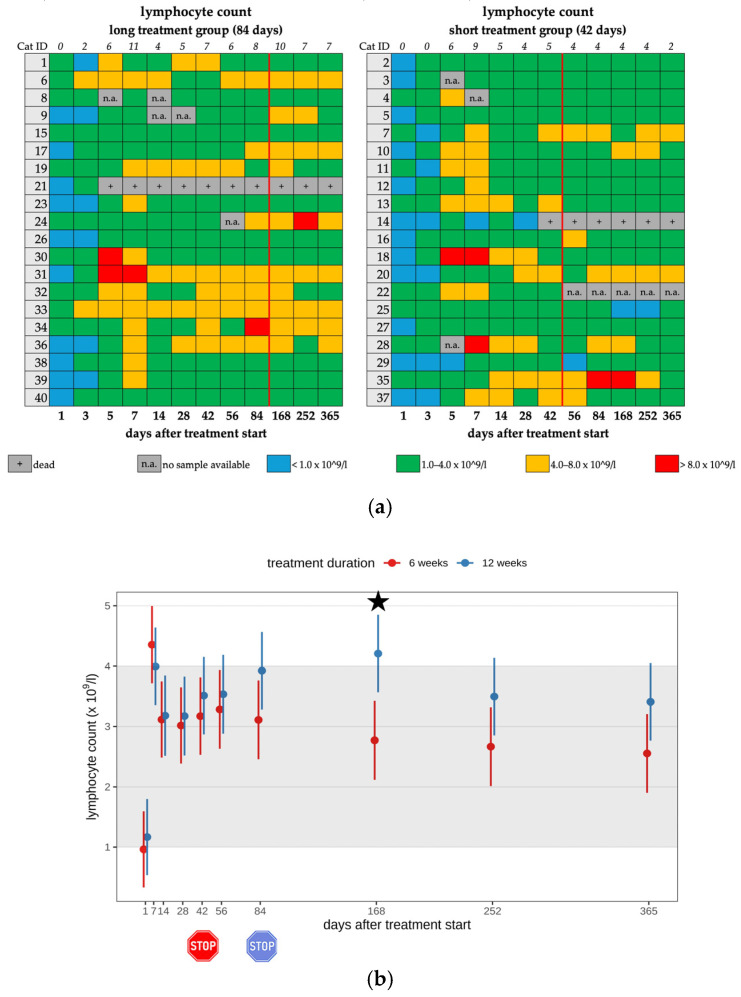
(**a**) Heatmap visualizing the lymphocyte count in cats throughout the study period in both groups until day 365 (comparison of treatment duration: 42 versus 84 days). Lymphocyte count < 1.0 × 10^9^/L (blue) is defined as lymphopenia, lymphocyte count of 1.0–4.0 × 10^9^/L (green) represents a normal value, lymphocyte count of 4.0–8.0 × 10^9^/L (orange) is defined as mild lymphocytosis, lymphocyte count of >8.0 × 10^9^/L (red) is defined as moderate lymphocytosis. The classification of lymphocytosis grades is based on the publication by Zuzzi-Krebitz et al., 2024 [6]. The red line indicates the end of the treatment period, and the line above the map represents the number of cats with lymphocytosis. (**b**) Timeline visualizing changes in lymphocyte counts in cats during the treatment with GS-441524 until day 42 (red) or day 84 (blue) and up to day 365 (comparison of treatment duration: 42 versus 84 days). The red stop sign marks the end of treatment of the short treatment group; the blue stop sign marks the end of treatment of the long treatment group. Figures show average predictive values and 95% confidence intervals of each parameter. Grey shading marks the reference interval of the parameters. Black asterisks indicate statistically significant differences in group comparison (*p* < 0.05) measured by robust linear mixed-effects model.

In twelve cats (12/26) in the short treatment group and fourteen cats (14/26) in the long treatment group, lymphocytosis (>4.0 × 10^9^/L) was present during treatment (26/40; 65.0%). Most cats (21/26) showed only mild lymphocytosis (4.0–7.9 × 10^9^/L) (Figure 3a,b). After treatment discontinuation, lymphocytosis was still present in 17 cats, with 7/17 cats of the short and 10/17 cats of the long treatment group (Table 2, Table 3 and Table 4). Cats that received short treatment had a significantly lower lymphocyte count after the end of treatment compared to cats treated longer. A significant difference was observed between the two treatment duration groups regarding the lymphocyte count on day 168 (*p* = 0.001). Cats treated for a longer treatment duration (84 days) showed significantly higher lymphocyte counts than those that had already terminated their antiviral treatment on day 42. This means that the longer treatment caused significantly higher lymphocyte counts than the short treatment. The cats with lymphocytosis did not show any clinical effects that were directly attributed to these hematological changes.

### 3.3. Eosinophilia

**Figure 4:**
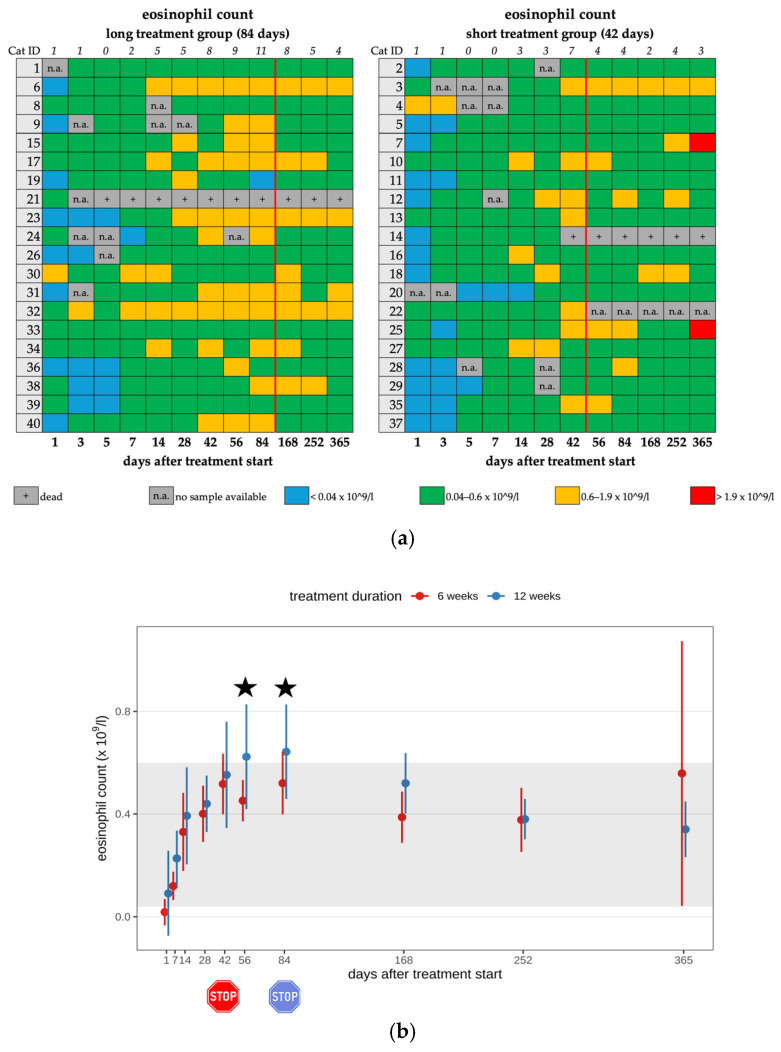
(**a**) Heatmap visualizing the eosinophil count in cats throughout the study period in both groups until day 365 (comparison of treatment duration: 42 versus 84 days). Eosinophil count < 0.04 × 10^9^/L (blue) is defined as eosinopenia, eosinophil count of 0.04–0.6 × 10^9^/L (green) represents a normal value, eosinophil count of 0.6–1.9 × 10^9^/L (orange) is defined as mild eosinophilia, and eosinophil count of >1.9 × 10^9^/L (red) is defined as moderate eosinophilia. The classification of eosinophilia grades is based on the publication by Zuzzi-Krebitz et al. (2024) [6]. The red line indicates the end of the treatment period, and the line above the map represents the number of cats with eosinophilia. (**b**) Timeline visualizing changes in eosinophil counts in cats during the treatment with GS-441524 until day 42 (red) or day 84 (blue) and up to day 365 (comparison of treatment duration: 42 versus 84 days). The red stop sign marks the end of treatment of the short treatment group; the blue stop sign marks the end of treatment of the long treatment group. Figures show average predictive values and 95% confidence intervals of each parameter. Grey shading marks the reference interval of the parameters. Black asterisks indicate statistically significant differences in group comparison (*p* < 0.05) measured by the median mixed-effects model.

Eleven cats (11/25) in the short treatment group and fourteen cats (14/25) in the long treatment group developed mild eosinophilia (0.06–1.9 × 10^9^/L) during their treatment course (25/40; 62.5%) (Figure 4a,b). After the end of treatment, only 16 cats had eosinophilia, with 8 of them in each treatment duration group (Table 2, Table 3 and Table 4). A significant difference was observed regarding the eosinophil count between the two treatment groups on day 56 (*p* = 0.02) and day 84 (*p* = 0.046). Cats treated for 84 days showed significantly higher eosinophil counts on days 56 and 84 (during their antiviral treatment) than cats in the short treatment group that had already terminated their antiviral treatment. In all cats with eosinophilia or diarrhea, fecal examinations were performed to exclude parasite infestation as a potential cause. In three cats (cat 7, cat 20, cat 35), *Giardia* spp. infection was detected during treatment with GS-441524 on days 14, 56, and 32, respectively. These cats were treated with fenbendazole. None of these cats had diarrhea. In cat 7, co-infection with *Toxocara* spp. infection was detected by fecal examination. This cat exhibited diarrhea during the preceding days (days 5–14). The cats with eosinophilia did not show any clinical effects that were directly attributed to these hematological changes.

### 3.4. Changes in Liver Enzyme Activity 

**Figure 5:**
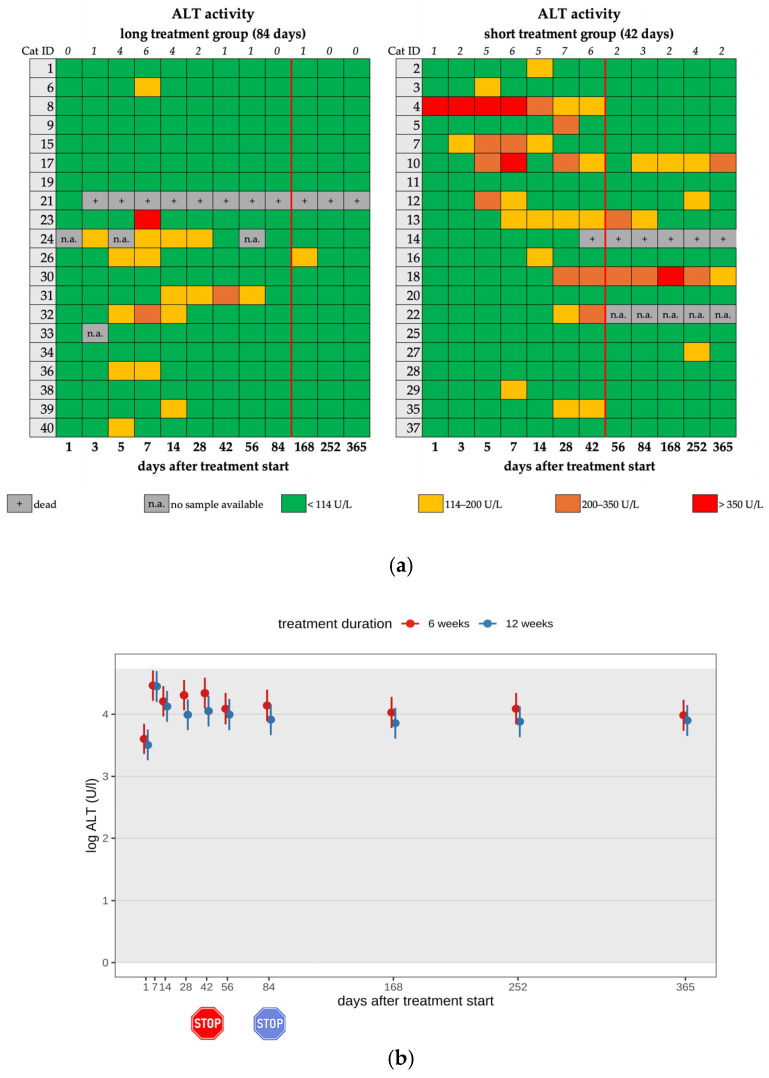
(**a**) Heatmap visualizing ALT activity in cats throughout the study period in both groups until day 365 (comparison of treatment duration: 42 versus 84 days). ALT activity < 114 U/L (green) represents a normal value, ALT activity of 114–200 U/L (yellow) represents a mild increase, ALT activity of 200–350 U/L (orange) represents a moderate increase, and ALT activity > 350 U/L (red) represents a severe increase. The red line indicates the end of the treatment period, and the line above the map represents the number of cats with increased ALT activity. (**b**) Timeline visualizing changes in ALT activity of the cats during the treatment with GS-441524 until day 42 (red) or day 84 (blue) and up to day 365 (comparison of treatment duration: 42 versus 84 days). The red stop sign marks the end of treatment of the short treatment group; the blue stop sign marks the end of treatment of the long treatment group. Figures show average predictive values and 95% confidence intervals of each parameter. Grey shading marks the reference interval of the parameters. There was no statistically significant difference in group comparison measured by the robust linear mixed-effects model. ALT activity was log-transformed to correct for strong skewness in its distribution.

An increase in ALT activity (>114 IU/L) was noted in 13 cats (13/22) of the short and 9 cats (9/22) of the long treatment group during their treatment course (22/40; 55.0%) (Figure 5a,b). In 7/22 cats, the increase in ALT activity was moderate (ALT 200–350 IU/L). In 3/22 cats, the increase was severe (ALT > 350 IU/L); therefore, treatment with silymarin (20 mg/kg, PO, q12h for 10 days, following 20 mg/kg, PO, q24h) was initiated until the liver enzyme activity returned to within the reference interval (RI) (Table 2, Table 3 and Table 4). An increase in ALT activity was seen in only six cats after treatment cessation, in five cats of the short and one cat of the long treatment group. One cat (cat 18) showed an increase in ALT activity from day 28 on (191–514 IU/L). Due to a further increase in ALT activity on day 102 (406 IU/L), treatment with silymarin was initiated. On day 145, ALT activity deteriorated further (514 IU/L); therefore, additional S-adenosyl-methionine (SAMe) (20 mg/kg, PO, q24h) was started. Treatment with silymarin and SAMe was stopped after the 365-day follow-up examination. During a re-check three months later, the ALT activity was only mildly elevated.

**Figure 6:**
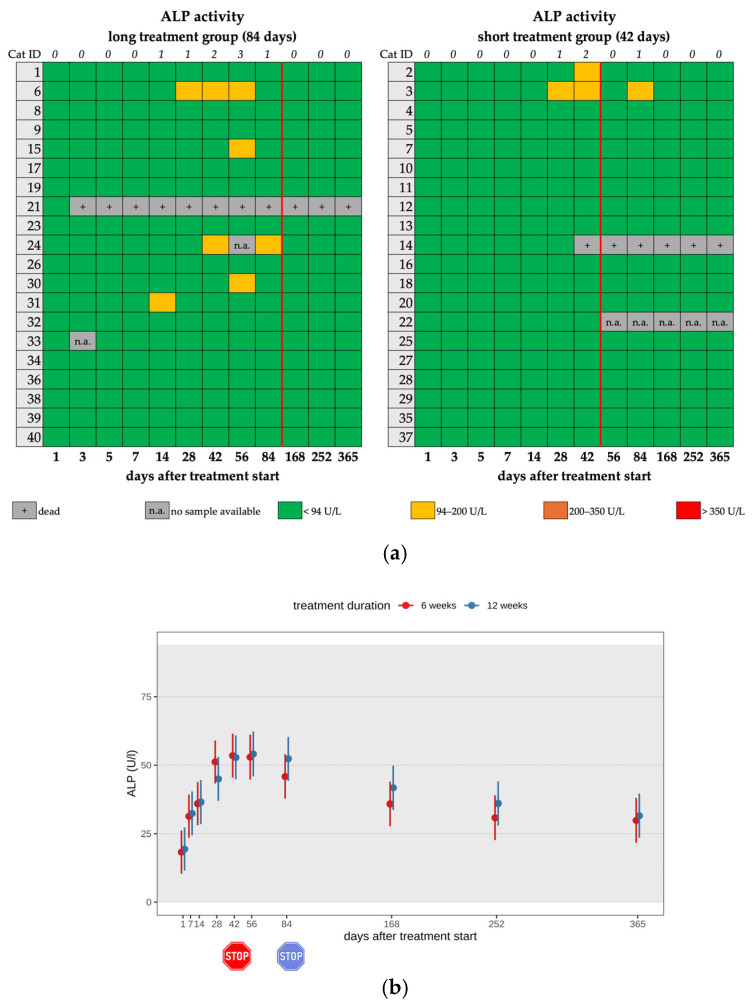
(**a**) Heatmap visualizing ALP activity in cats throughout the study period in both groups until day 365 (comparison of treatment duration: 42 versus 84 days). ALP activity < 94 U/L (green) represents a normal value, ALP activity of 94–200 U/L (yellow) represents a mild increase, ALP activity of 200–350 U/L (orange) represents a moderate increase, and ALP activity > 350 U/L (red) represents a severe increase. The red line indicates the end of the treatment period, and the line above the map represents the number of cats with increased ALP activity. (**b**) Timeline visualizing changes in ALP activity of the cats during the treatment with GS-441524 until day 42 (red) or day 84 (blue) and up to day 365 (comparison of treatment duration: 42 versus 84 days). The red stop sign marks the end of treatment of the short treatment group; the blue stop sign marks the end of treatment of the long treatment group. Figures show average predictive values and 95% confidence intervals of each parameter. Grey shading marks the reference interval of the parameters. There was no statistically significant difference in group comparison measured by the robust linear mixed-effects model.

A mild increase in ALP activity (94–200 IU/L) was seen in 7/40 (17.5%) cats, with a maximum value of 137 IU/L during the treatment; only one cat (cat 3) of the short treatment showed a mild ALP increase after the end of treatment (day 84) (Figure 6a,b; Table 2, Table 3 and Table 4). All cats with ALP elevation were younger than one year.

There was no significant difference in group comparison regarding the elevation of liver enzyme activity; cats showed an increase in liver enzyme activity independent of whether they were treated for 42 or 84 days.

### 3.5. Changes in Renal Function Parameters 

**Figure 7:**
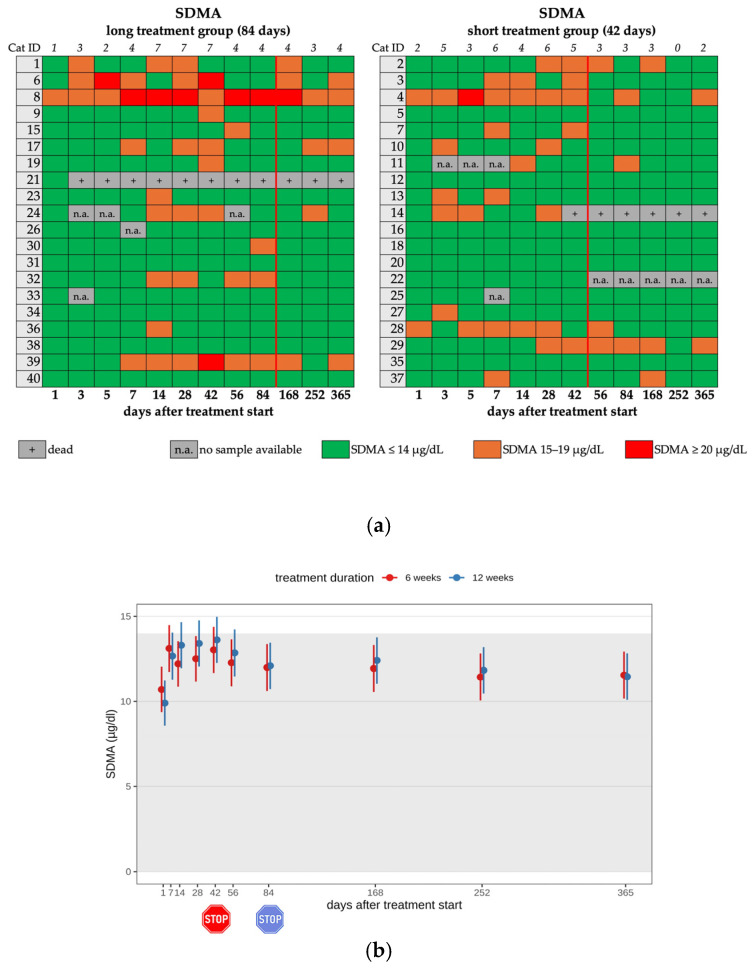
(**a**) Heatmap visualizing the SDMA levels in cats throughout the study period in both groups until day 365 (comparison of treatment duration: 42 versus 84 days). SDMA levels ≤ 14 µg/dL (green) represent a normal value, SDMA levels of 15–19 µg/dL (orange) represent a mild increase, SDMA levels of ≥20 µg/dL (red) represent a moderate increase. The red line indicates the end of the treatment period, and the line above the map represents the number of cats with increased SDMA levels. (**b**) Timeline visualizing changes in SDMA levels in cats during the treatment with GS-441524 until day 42 (red) or day 84 (blue) and after discontinuation of treatment up to day 365 (comparison of treatment duration: 42 versus 84 days). The red stop sign marks the end of treatment of the short treatment group; the blue stop sign marks the end of treatment of the long treatment group. Figures show average predictive values and 95% confidence intervals of each parameter. Grey shading marks the reference interval of the parameters. There was no statistically significant difference in group comparison measured by the linear mixed-effects model.

Increased SDMA levels (>14 µg/dL) were seen in 12 cats (12/25) in the short and 13 cats (13/25) in the long treatment group during treatment (25/40; 62.5%). Most cats (21/25) showed only a mild increase (Figure 7a,b). In 3 of these 25 cats, SDMA levels were already elevated on day 1 before the treatment start with GS-441524. After completion of the treatment, twelve cats had increased SDMA levels, with six of them in each treatment group (Table 2, Table 3 and Table 4). On day 365, six cats still showed increased SDMA levels. There was no significant difference between treatment groups throughout the entire study period.

**Figure 8:**
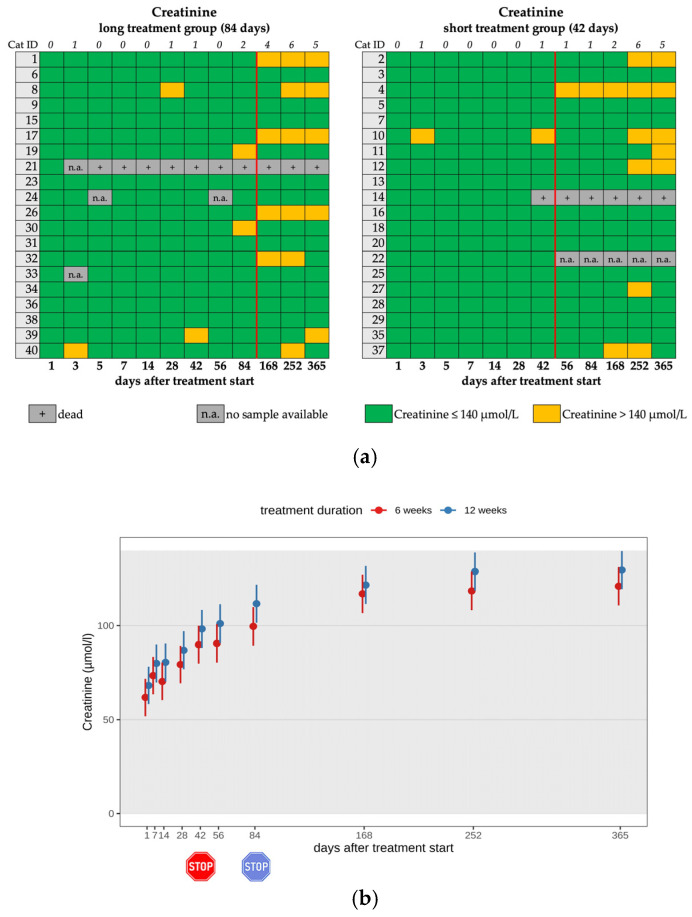
(**a**) Heatmap visualizing creatinine levels in cats throughout the study period in both groups until day 365 (comparison of treatment duration: 42 versus 84 days). Creatinine levels < 140 µmol/L (green) represent a normal value (according to the IRIS guidelines). Creatinine levels > 140 µmol/L (yellow) represent a mild increase. The red line indicates the end of the treatment period, and the line above the map represents the number of cats with increased creatinine levels. (**b**) Timeline visualizing changes in creatinine levels in cats during the treatment with GS-441524 until day 42 (red) or day 84 (blue) and up to day 365 (comparison of treatment duration: 42 versus 84 days). The red stop sign marks the end of treatment of the short treatment group; the blue stop sign marks the end of treatment of the long treatment group. Figures show average predictive values and 95% confidence intervals of each parameter. Grey shading marks the reference interval of the parameters. There was no statistically significant difference in group comparison measured by the robust linear mixed-effects model.

On day 1, creatinine levels were unremarkable in all cats. During the study period, an increase in creatinine levels was noted in almost all cats. However, in most cats, the increase was within the reference interval until day 84 (Figure 8a,b). After treatment termination, seven cats had creatinine levels ≥ 140 µmol/l and a concurrent USG < 1.035, categorizing them as cats with renal azotemia [24]. Postrenal causes of azotemia were excluded based on ultrasonography findings. Interestingly, only one cat (cat 39) showed renal azotemia and elevated SDMA levels simultaneously, as well as an increased UPC (>0.4) on day 365. This cat had been diagnosed with bilateral nephrolithiasis prior to the initiation of antiviral treatment.

UPC was measured in 35 cats at different time points during treatment with GS-441524; a UPC > 0.4 (RI: <0.4) was seen in nine cats during antiviral treatment and in five cats after treatment termination, with clean sediments present in all cases. Two cats had persistent proteinuria (with a maximum value of 7.78) without azotemia (cats 28 and 36) and were treated with telmisartan (1 mg/kg, PO, q24h).

In addition to the ultrasonographic findings commonly associated with FIP, pyelectasis ≥ 3.5 mm, which is considered potentially abnormal [25], was diagnosed in two cats (cats 19 and 39) during the study period. Cat 19 (long treatment group) showed negligible pyelectasis (1.7 mm) in the left kidney on day 1 (but received 3 mL/kg/h fluid therapy the day before). Due to deterioration on day 84 (9.0 mm), treatment with terazosin (0.5 mg/cat, PO, q24h) was given until improvement of the renal pelvis dilation eight weeks later. No uroliths could be found by ultrasonography or radiography. Cat 39 (also long treatment group) already had nephrolithiasis in both kidneys before starting the antiviral treatment; on day 42, severe pyelectasis (10 mm) of the left kidney and ureteral obstruction were diagnosed by ultrasonography; therefore, a subcutaneous ureteral bypass (SUB) device was placed. The uroliths obtained during surgery were analyzed and identified as calcium oxalate.

### 3.6. Hemolysis

Three cats developed specific forms of anemia during antiviral treatment. Heinz bodies were seen in erythrocytes in two cats during treatment. Cat 8 (long treatment group) showed moderate Heinz body anemia (hematocrit 27.4%, RI: 33.0–44.0%), with 19.0% of red blood cells containing Heinz bodies on day 7. Cat 13 (short treatment group) showed mild Heinz body anemia (hematocrit 32.0%), with 6.3% of red blood cells containing Heinz bodies on day 14. These cats were treated with SAMe 20 mg/kg, PO, q24h, and the hematocrit was within the reference interval at the subsequent regular control examination.

Immune-mediated hemolytic anemia (IMHA) was diagnosed in cat 25 (short treatment group). The cat received a whole blood transfusion on the day of IMHA diagnosis (day 9), and treatment with prednisolone (2 mg/kg, PO, q24h) was initiated. On day 42, the hematocrit was within the reference interval (33.0%).

## 4. Discussion

This is the first prospective, controlled study examining unexpected observations during and after treatment with GS-441524 in cats with FIP with effusion and comparing two groups with different treatment durations (long vs. short; 84 vs. 42 days). These unexpected observations can occur during and after treatment with GS-441524, with most of them improving during the follow-up period (days 168–365). Some unexpected observations during treatment with GS-441524 have already been reported in recently published studies [2,4,9,17,18,20]. However, so far, they have not been described in detail or compared in relation to the treatment duration. Therefore, the aim was to definitively determine whether GS-441524 in cats with FIP should only be administered as briefly as possible and necessary.

In total, 26/40 cats developed mild to moderate lymphocytosis during the antiviral treatment with GS-441524. Initially, most cats had lymphopenia, which is a common sign in cats with FIP and likely due to increased apoptosis from pro-apoptotic factors released by infected macrophages [26,27,28]. Similar findings could be seen in 18 cats with FIP that were treated with GS-441524 by the same research group [4,5]; interestingly, also in coronavirus disease 2019 (COVID-19) patients treated with remdesivir [29], the prodrug of GS-441524, and in cats treated with GC376 [30]. The occurrence of lymphocytosis following antiviral treatment was generally interpreted in those earlier studies as a sign of recovery rather than a side effect [4,31].

Further analysis of the lymphocytosis of some of these previous 18 cats showed that this increase in lymphocyte count was mainly due to higher numbers of T-helper cells (*p* < 0.05) and B-cells (*p* < 0.001), while cytotoxic T-cells remained unchanged. It was discussed that the rise in T-cells could reflect regulatory immune activity, and the B-cell increase could result from reduced FCoV-induced apoptosis. It was hypothesized that these findings could indicate that the observed immune response supports the efficacy of antiviral treatment [31,32]. However, in the present study, a comparison between the two groups with different treatment durations showed that long-term-treated cats (84 days) had significantly higher lymphocyte counts on day 168 compared to those treated for only 42 days. If lymphocytosis were solely a marker of recovery, its occurrence would be expected to be of a similar length of time, regardless of the duration of the antiviral treatment. The fact that a longer treatment duration was associated with prolonged lymphocytosis suggests that this might also represent a true side effect of the antiviral treatment. Another explanation for the prolonged lymphocytosis observed after treatment cessation could be that GS-441524 also has an additional immunostimulatory effect. Further studies are warranted to determine the underlying mechanism and to determine if the lymphocyte stimulatory effect is rather beneficial or has negative effects, such as a higher risk for lymphoma development.

Persistent lymphadenomegaly, observed during long-term follow-up of cats successfully treated with GS-441524 [5], might thus be associated with an ongoing immune overstimulation, which could potentially contribute to the development of lymphoma. Lymphoma has already been described in cats treated with GS-441524 [33]. Other possible reasons for the lymphocytosis, such as young age or stress, seem less likely, as the age distribution and stress levels were comparable in both groups.

Eosinophilia occurred in 25/40 cats in the present study during the antiviral treatment, which is comparable to other studies (52.0% [17]–61.1% [4]). It has been suggested that eosinophilia in humans [34] as well as in cats [4] is a sign of recovery from COVID-19 and FIP, respectively. However, in the present study, long-term-treated cats (84 days) showed significantly higher levels of eosinophil counts (on days 56 and 84) than the cats that were treated for only 42 days. There was no association of eosinophilia with the occurrence of lymphocytosis. It was hypothesized that GS-441524 possibly activates the complement system, thereby chemotactically attracting eosinophils [6]. An alternative mechanism could be the effect of Interleukin-5 (IL-5), which leads to increased production and prolonged survival of the eosinophils [35]. However, the fact that eosinophilia occurs more often in the cats that were treated for a longer treatment duration suggests a possible association with GS-441524 treatment. Other medications have also been reported to induce nonspecific eosinophilia [36]. However, interestingly, an equal number of cats in both treatment groups exhibited eosinophilia even after the completion of the antiviral treatment, which could suggest a persistent immune response in these cats. Other causes for eosinophilia, such as parasite infestation and allergies, are unlikely. In the context of elevated peripheral blood cell counts, such as eosinophilia and lymphocytosis, it is also essential to consider whether this increase results from enhanced hematopoietic activity (e.g., in the bone marrow and thymus), prolonged cellular survival mediated by an anti-apoptotic mechanism, or impaired egress into tissues, potentially caused by altered expression of adhesion molecules. Further studies on the occurrence and relevance of lymphocytosis and eosinophilia in cats treated with antiviral drugs are needed.

Further changes in blood analytes included increased liver enzyme activity (ALT, ALP). However, there was no significant difference in the group comparison, and normalization into the reference interval was observed in most cats at the latest after treatment cessation. A similar phenomenon was also observed in cats in other treatment studies [4,14,17]. It was suspected that this increase was most likely due to the metabolism of GS-441524 in the liver, indicating a mild and only temporarily occurring side effect of the antiviral therapy. However, in some cases, the elevation persisted even beyond the end of treatment. Coggins and colleagues (2023) discussed whether this persistent increase might be attributed to lasting liver damage caused by FIP rather than to the antiviral treatment [17], as FIP can cause liver cell damage [37], such as hepatic lesions, including perihepatitis or granulomatous-necrotizing inflammation [38,39]. However, a pharmacokinetic study showed that GS-441524 can accumulate in the liver [40], and hepatotoxic effects of remdesivir, its prodrug, have also been published [41]. Therefore, it remains challenging to determine whether the observed changes in the cats were induced by GS-441524 or resulted from FIP itself. The increase in ALP levels in the cats of the current study is negligible, as all of these cats were younger than one year, and in this age group, elevated levels are considered physiologically normal due to ongoing growth and bone metabolism [42,43].

In the present study, there was a continuous increase in creatinine levels; however, it was within the reference interval during the study period in all cats, which can be explained by a gain in muscle mass due to growth and recovery from FIP [44]. After treatment termination, renal azotemia was observed in seven cats; however, the USG fluctuated and, in some cases, returned to normal values. There was no significant difference between the treatment groups. Interestingly, elevations of SDMA above 14 µg/dl were seen in 62.5% (25/40) of the cats during the treatment period. This contrasts with previous research, in which an increase in SDMA during antiviral treatment of cats with FIP was reported as a rare finding [2,4,9]. Most of the cats with elevated SDMA in the present study had concentrations between 15 and 19 µg/dl. In two cats, SDMA was persistently elevated to >20 µg/dl. These cats (cats 4 and 8) were among the five cats with a diagnosis of chronic kidney disease on day 365. Therefore, mild elevations of SDMA during treatment seem to be a common effect and are not necessarily associated with the development of chronic kidney disease, at least within the study period of 365 days. The biological or analytical variability of SDMA should also be considered [45]. It remains unclear whether this mild elevation of SDMA reflects a decline in glomerular filtration rate (GFR), represents its biological variation as previously described [46], or was caused by a different mechanism. In addition, 2/40 cats had persistent renal proteinuria without azotemia, which also indicates renal disease. The degree of proteinuria with a UPC up to 7.78 was strongly suggestive of a glomerular origin. As UPC was not measured before treatment start, it is difficult to assess the effect of medication, but as FIP has been associated with membranoproliferative glomerulonephritis [47], it is more likely that this was not a drug-associated side effect but rather caused by FIP itself. Altogether, 7/40 cats developed either non-proteinuric kidney disease (5/7) or proteinuric kidney disease (2/7) during the study period, 5/40 of them after treatment. Monitoring these cats after treatment is therefore important for identifying and treating renal toxicity.

A previous case report described urolithiasis in two cats with FIP during treatment with illegally produced GS-441524; the stone analysis confirmed a composition of 98% GS-441524 [20,48]. In contrast, cat 39 had already been diagnosed with nephrolithiasis before the treatment with GS-441524, and the uroliths obtained during surgery were analyzed as calcium oxalate. Thus, no causal relationship between treatment with GS-441524 and urolith formation in cat 39 could be established in the present study. Urolithiasis in the two cats [20] of the published case report was likely associated with a higher administered dosage, as unlicensed formulations have been shown to contain, on average, 39% more GS-441524 than indicated on the label [49]. Consequently, this does not appear to occur when GS-441524 is administered at an appropriate dosage.

Diarrhea was observed in 25/40 cats during treatment with GS-441524, representing a higher incidence compared to previous treatment studies with oral GS-441524 where no [4], two (2/9, 22.2%) [18], or four (4/203, 2.0%) cats [16] were affected. In the previous study by the same research group, no cats showed diarrhea; in this study, however, another illegally produced multi-component preparation (Xraphconn^®^ (Mutian Life Sciences Limited, Nantong, China)) was used [4], which also contained herbal preparations in addition to GS-441524. These additional ingredients might have had a positive effect on the intestinal microbiome of these cats. Another potential explanation for the frequently observed diarrhea in the present study, when compared to the other study, is an increased administered oral dosage of the active substance [4], which might alter the composition of the gut flora. Alternatively, FIP itself could lead to an altered gut flora before the initiation of treatment, which might be further influenced by the oral GS-441524. In comparison, it has already been reported in human medicine that diarrhea affects approximately 10.0–20.0% of COVID-19 patients [50]. Furthermore, it is described that severe acute respiratory syndrome coronavirus type 2 (SARS-CoV-2) infection is associated with an alteration of the intestinal microbiome [51]. Therefore, examinations of the feline intestinal microbiome before, during, and after treatment with varying oral dosages of GS-441524 should be considered in future studies. Additionally, it is important to consider other potential causes of diarrhea, such as dietary changes or the administration of antibiotics. Antibiotics were administered to 29/40 cats during antiviral treatment due to suspicion of a secondary bacterial infection, and 19 of these cats developed diarrhea. However, as diarrhea was no longer observed after treatment termination, and some owners also reported that stool consistency directly improved when treatment with GS-441524 was stopped, it is likely that diarrhea was indeed a treatment-related side effect.

Limitations of this study included that not every parameter in every cat could be measured, such as UPC ratios, due to the inability to perform cystocentesis, e.g., due to thrombocytopenia or small bladder size. Furthermore, testing for additional viruses, such as the feline morbillivirus or hepatitis B-like viruses, was not performed, which might also influence hepatic or renal function.

As the included cats were owned cats living in residential or other environments, various unmeasured exposures could have been possible. Therefore, it could be argued that some of these unexpected clinical and laboratory observations, particularly in the months following the conclusion of treatment, might have been due to other factors, including individual cat genetics, diet, and environment. For example, diet could impact creatinine levels or contribute to the development of diarrhea.

In this study, it is not possible to definitively determine whether these unexpected observations are caused by the antiviral treatment, are a consequence of FIP, or are due to other concurrent subclinical co-infections or immune-mediated processes. Future studies with larger cohorts and controlled conditions are necessary to clarify these relationships.

## 5. Conclusions

In conclusion, the present study demonstrated that various unexpected clinical and laboratory observations, such as diarrhea, lymphocytosis, eosinophilia, and an increase in liver enzyme activities or renal function parameters, can occur during and after GS-441524 treatment. As there were significantly higher values in cats that were treated for 84 days compared to cats with a 42-day treatment, lymphocytosis and eosinophilia might be categorized as side effects of treatment with GS-441524. A shorter treatment duration is indicated, as it reduces the risk of side effects and possible long-term consequences. This study once more shows that it is highly recommended and essential that the antiviral treatment of FIP be managed by veterinarians to ensure the early identification of such unexpected clinical and laboratory observations and to facilitate appropriate management.

## Figures and Tables

**Table 1:** Signalment of cats with FIP treated with GS-441524 for 42 or 84 days, including age, breed, sex, and number of cats with partner cats.

Parameters	Short Treatment Group	Long Treatment Group
	**Age**	
Range of Age in Months	6.4–98.6	5.1–116.3
Median Age in Months	12.3	14.95
	**Breeds**	
Domestic Shorthair	11 (55.0%)	5 (25.0%)
British Shorthair	3 (15.0%)	5 (25.0%)
Maine Coon	2 (10.0%)	1 (5.0%)
Siamese	1 (5.0%)	0 (0.0%)
Holy Birman	0 (0.0%)	1 (5.0%)
Scottish Fold/Straight	1 (5.0%)	1 (5.0%)
Somali	0 (0.0%)	1 (5.0%)
Mixed Breed	2 (10.0%)	3 (15.0%)
Exotic Shorthair	0 (0.0%)	1 (5.0%)
Oriental Shorthair	0 (0.0%)	1 (5.0%)
Ragdoll	0 (0.0%)	1 (5.0%)
	**Sex**	
Male	14 (70.0%)	17 (85.0%)
(neutered)	11/14 (78.6%)	10/17 (58.8%)
Female	6 (30.0%)	3/20 (15.0%)
(neutered)	1/6 (16.7%)	1/3 (33.3%)
Partner Cats?	17/20 (85.0%)	13/20 (65.0%)

**Table 2:** Unexpected clinical and laboratory observations in 20/40 cats with FIP during the short treatment duration of 42 days and after treatment, grades of the unexpected observations, and respective symptomatic treatment.

Unexpected Clinical and Laboratory Observations	Number of Cats		Grade	Symptomatic Treatment
**During Treatment (until Day 42)**				
Diarrhea ^1^	14/20	11/14	mild	
		3/14	severe	probiotics, fluid therapy
Lymphocytosis ^2^	12/20	10/12	mild	none
		2/12	moderate	
Eosinophilia ^3^	11/20	11/11	mild	none
Increased ALT ^4^	13/20	6/13	mild	
		5/13	moderate	
		2/13	severe	silymarin ^8^, SAMe ^9^
Increased ALP ^5^	2/20	2/2	mild	none
Increased SDMA ^6^	12/20	11/12	mild	none
		1/12	moderate	
**After Treatment (Days 56, 84, 168, 252, 365)**				
Lymphocytosis	7/18	6/7	mild	none
		1/7	moderate	
Eosinophilia	8/18	6/8	mild	none
		2/8	moderate	
Increased ALT	5/18	1/5	mild	
		3/5	moderate	
		1/5	severe	silymarin
Increased ALP	1/18	1/1	mild	none
Increased SDMA	6/18	6/6	mild	none
Renal Azotemia ^7^	4/18	4/4	mild	dietary changes

One cat of the 42-day treatment group and one cat of the 84-day treatment group died before the completion of antiviral treatment. Additionally, one cat of the 42-day treatment group was lost to follow-up. ^1^ Diarrhea: mild (Purina fecal score of 5 or 6), severe (Purina fecal score of 7), ^2^ lymphocytosis: mild (4–7.9 × 10^9^/L), moderate (8–15 × 10^9^/L), severe (>15 × 10^9^/L); ^3^ eosinophilia: mild (0.6–1.9 × 10^9^/L), moderate (2–10 × 10^9^/L), severe (>10 × 10^9^/L); ^4^ increased alanine aminotransferase (ALT) activity: mild (ALT 114–200 IU/L), moderate (ALT 200–350 IU/L), severe (ALT > 350 IU/L); ^5^ increased alkaline phosphatase (ALP) activity: mild (ALP 94–200 IU/L), moderate (ALP 200–350 IU/L), severe (ALP > 350 IU/L); ^6^ increased SDMA: mild (SDMA 15–19 µg/dL), moderate (SDMA ≥ 20 µg/dL); ^7^ renal azotemia: increased creatinine in combination with low urine specific gravity < 1.035; ^8^ S-adenosyl-methionine 20 mg/kg, PO, q24h; ^9^ silymarin 20 mg/kg, PO, q12h for 10 days followed by 20 mg/kg, PO, q24h for treatment of increased liver enzyme activities.

**Table 3:** Unexpected clinical and laboratory observations in 20/40 cats with FIP during the long treatment duration of 84 days, and after treatment, grades of the unexpected observations, and respective symptomatic treatment.

Unexpected Clinical and Laboratory Observations	Number of Cats		Grade	Symptomatic Treatment
**During Treatment (until Day 84)**				
Diarrhea ^1^	11/20	9/11	mild	
		2/11	severe	probiotics, fluid therapy
Lymphocytosis ^2^	14/20	11/14	mild	none
		3/14	moderate	
Eosinophilia ^3^	14/20	14/14	mild	none
Increased ALT ^4^	9/20	6/9	mild	
		2/9	moderate	
		1/9	severe	silymarin ^8^, SAMe ^9^
Increased ALP ^5^	5/20	5/5	mild	none
Increased SDMA ^6^	13/20	10/13	mild	none
		3/13	moderate	
**After Treatment (Days 168, 252, 365)**				
Lymphocytosis	10/19	9/10	mild	none
		1/10	moderate	
Eosinophilia	8/19	8/8	mild	none
Increased ALT	1/19	1/1	mild	
Increased SDMA	6/19	5/6	mild	none
		1/6	moderate	
Renal Azotemia ^7^	3/19	3/3	mild	dietary changes

One cat of the 42-day treatment group and one cat of the 84-day treatment group died before the completion of antiviral treatment. Additionally, one cat of the 42-day treatment group was lost to follow-up. ^1^ Diarrhea: mild (Purina fecal score of 5 or 6), severe (Purina fecal score of 7), ^2^ lymphocytosis: mild (4–7.9 × 10^9^/L), moderate (8–15 × 10^9^/L), severe (>15 × 10^9^/L); ^3^ eosinophilia: mild (0.6–1.9 × 10^9^/L), moderate (2–10 × 10^9^/L), severe (>10 × 10^9^/L); ^4^ increased alanine aminotransferase (ALT) activity: mild (ALT 114–200 IU/L), moderate (ALT 200–350 IU/L), severe (ALT > 350 IU/L); ^5^ increased alkaline phosphatase (ALP) activity: mild (ALP 94–200 IU/L), moderate (ALP 200–350 IU/L), severe (ALP > 350 IU/L); ^6^ increased SDMA: mild (SDMA 15–19 µg/dL), moderate (SDMA ≥ 20 µg/dL); ^7^ renal azotemia: increased creatinine in combination with low urine specific gravity < 1.035; ^8^ S-adenosyl-methionine 20 mg/kg, PO, q24h; ^9^ silymarin 20 mg/kg, PO, q12h for 10 days followed by 20 mg/kg, PO, q24h for treatment of increased liver enzyme activities.

**Table 4:** Unexpected clinical and laboratory observations in cats with FIP during and after GS-441524 treatment divided into the two treatment duration groups and number of cats in which these unexpected clinical and laboratory observations never occurred. Some cats exhibited unexpected clinical and laboratory observations both during and after treatment.

Unexpected Clinical and Laboratory Observations	Occurred During Treatment		Occurred After Treatment		Never Occurred	
	42-Day Treatment	84-Day Treatment	42-Day Treatment	84-Day Treatment	42-Day Treatment	84-Day Treatment
Diarrhea ^1^	14/20	11/20	0/18	0/19	6/20	9/20
Lymphocytosis	12/20	14/20	7/18	10/19	7/20	5/20
Eosinophilia	11/20	14/20	8/18	8/19	7/20	6/20
Increased ALT ^2^	13/20	9/20	5/18	1/19	6/20	11/20
Increased ALP ^3^	2/20	5/20	1/18	0/19	18/20	15/20
Increased SDMA ^4^	12/20	13/20	6/18	6/19	8/20	7/20
Renal Azotemia ^5^	0/20	0/20	4/18	3/19	15/20	16/20

One cat of the 42-day treatment group and one cat of the 84-day treatment group died before the completion of antiviral treatment. Additionally, one cat of the 42-day treatment group was lost to follow-up. ^1^ Diarrhea: mild (Purina fecal score of 5 or 6), severe (Purina fecal score of 7), ^2^ increased alanine aminotransferase (ALT) activity; ^3^ increased alkaline phosphatase (ALP) activity; ^4^ increased symmetric dimethylarginine (SDMA); ^5^ renal azotemia: increased creatinine in combination with low urine specific gravity < 1.035.

## Data Availability

The authors confirm that the datasets analyzed during the study are available from the corresponding author upon reasonable request.

## Abbreviations

The following abbreviations are used in this manuscript:

LMU	Ludwig-Maximilians University
FIP	feline infectious peritonitis
ALT	alanine aminotransferase
ALP	alkaline phosphatase
SDMA	symmetric dimethylarginine
RT-qPCR	reverse transcription quantitative polymerase chain reaction
FeLV	feline leukemia virus
FIV	feline immunodeficiency virus
UK	United Kingdom
PO	per os
q24h	every 24 h
FCoV	feline coronavirus
USG	urine specific gravity
UPC	urine protein creatinine ratio
RLMER	robust mixed-effects linear model
ICC	intraclass correlation coefficient
RMSE	root mean squared error
q12h	every 12 h
SAMe	S-adenosyl-methionine
RI	reference interval
SUB	subcutaneous ureteral bypass
IMHA	immune-mediated hemolytic anemia
COVID-19	coronavirus disease 2019
IL-5	interleukin-5
GFR	glomerular filtration rate
SARS-CoV-2	severe acute respiratory syndrome coronavirus type 2

## References

[1] Sparkes A.H., Gruffydd-Jones T.J., Harbour D.A. (1991). Feline infectious peritonitis: A review of clinicopathological changes in 65 cases, and a critical assessment of their diagnostic value. Vet. Rec..

[2] Pedersen N.C., Perron M., Bannasch M., Montgomery E., Murakami E., Liepnieks M., Liu H. (2019). Efficacy and safety of the nucleoside analog GS-441524 for treatment of cats with naturally occurring feline infectious peritonitis. J. Feline Med. Surg..

[3] Amirian E.S., Levy J.K. (2020). Current knowledge about the antivirals remdesivir (GS-5734) and GS-441524 as therapeutic options for coronaviruses. One Health.

[4] Krentz D., Zenger K., Alberer M., Felten S., Bergmann M., Dorsch R., Matiasek K., Kolberg L., Hofmann-Lehmann R., Meli M.L. (2021). Curing cats with feline infectious peritonitis with an oral multi-component drug containing GS-441524. Viruses.

[5] Zwicklbauer K., Krentz D., Bergmann M., Felten S., Dorsch R., Fischer A., Hofmann-Lehmann R., Meli M.L., Spiri A.M., Alberer M. (2023). Long-term follow-up of cats in complete remission after treatment of feline infectious peritonitis with oral GS-441524. J. Feline Med. Surg..

[6] Zuzzi-Krebitz A.M., Buchta K., Bergmann M., Krentz D., Zwicklbauer K., Dorsch R., Wess G., Fischer A., Matiasek K., Hönl A. (2024). Short treatment of 42 days with oral GS-441524 results in equal efficacy as the recommended 84-day treatment in cats suffering from feline infectious peritonitis with effusion—A prospective randomized controlled study. Viruses.

[7] Krentz D., Zwicklbauer K., Felten S., Bergmann M., Dorsch R., Hofmann-Lehmann R., Meli M.L., Spiri A.M., von Both U., Alberer M. (2022). Clinical follow-up and postmortem findings in a cat that was cured of feline infectious peritonitis with an oral antiviral drug containing GS-441524. Viruses.

[8] Murphy B.G., Perron M., Murakami E., Bauer K., Park Y., Eckstrand C., Liepnieks M., Pedersen N.C. (2018). The nucleoside analog GS-441524 strongly inhibits feline infectious peritonitis (FIP) virus in tissue culture and experimental cat infection studies. Vet. Microbiol..

[9] Addie D.D., Covell-Ritchie J., Jarrett O., Fosbery M. (2020). Rapid resolution of non-effusive feline infectious peritonitis uveitis with an oral adenosine nucleoside analogue and feline interferon omega. Viruses.

[10] Dickinson P.J., Bannasch M., Thomasy S.M., Murthy V.D., Vernau K.M., Liepnieks M., Montgomery E., Knickelbein K.E., Murphy B., Pedersen N.C. (2020). Antiviral treatment using the adenosine nucleoside analogue GS-441524 in cats with clinically diagnosed neurological feline infectious peritonitis. J. Vet. Intern. Med..

[11] Jones S., Novicoff W., Nadeau J., Evans S. (2021). Unlicensed GS-441524-like antiviral therapy can be effective for at-home treatment of feline infectious peritonitis. Animals.

[12] Katayama M., Uemura Y. (2021). Therapeutic effects of mutian(^®^) Xraphconn on 141 client-owned cats with feline infectious peritonitis predicted by total bilirubin levels. Vet. Sci..

[13] Lv J., Bai Y., Wang Y., Yang L., Jin Y., Dong J. (2022). Effect of GS-441524 in combination with the 3C-like protease inhibitor GC376 on the treatment of naturally transmitted feline infectious peritonitis. Front. Vet. Sci..

[14] Green J., Syme H., Tayler S. (2023). Thirty-two cats with effusive or non-effusive feline infectious peritonitis treated with a combination of remdesivir and GS-441524. J. Vet. Intern. Med..

[15] Yin Y., Li T., Wang C., Liu X., Ouyang H., Ji W., Liu J., Liao X., Li J., Hu C. (2021). A retrospective study of clinical and laboratory features and treatment on cats highly suspected of feline infectious peritonitis in Wuhan, China. Sci. Rep..

[16] Taylor S.S., Coggins S., Barker E.N., Gunn-Moore D., Jeevaratnam K., Norris J.M., Hughes D., Stacey E., MacFarlane L., O’Brien C. (2023). Retrospective study and outcome of 307 cats with feline infectious peritonitis treated with legally sourced veterinary compounded preparations of remdesivir and GS-441524 (2020–2022). J. Feline Med. Surg..

[17] Coggins S.J., Norris J.M., Malik R., Govendir M., Hall E.J., Kimble B., Thompson M.F. (2023). Outcomes of treatment of cats with feline infectious peritonitis using parenterally administered remdesivir, with or without transition to orally administered GS-441524. J. Vet. Intern. Med..

[18] Cosaro E., Pires J., Castillo D., Murphy B.G., Reagan K.L. (2023). Efficacy of oral remdesivir compared to gs-441524 for treatment of cats with naturally occurring effusive feline infectious peritonitis: A blinded, non-inferiority study. Viruses.

[19] Addie D.D., Silveira C., Aston C., Brauckmann P., Covell-Ritchie J., Felstead C., Fosbery M., Gibbins C., Macaulay K., McMurrough J. (2022). Alpha-1 acid glycoprotein reduction differentiated recovery from remission in a small cohort of cats treated for feline infectious peritonitis. Viruses.

[20] Allinder M., Tynan B., Martin C., Furbish A., Austin G., Bartges J., Lourenco B.N. (2024). Uroliths composed of antiviral compound gs-441524 in 2 cats undergoing treatment for feline infectious peritonitis. J. Vet. Intern. Med..

[21] Hartmann K., Kuffer M. (1998). Karnofsky’s score modified for cats. Eur. J. Med. Res..

[22] Purina Fecal Scoring Chart. https://vetcentre.purina.co.uk/sites/default/files/2021-11/Faecal%20scoring%20chart_general%20use.pdf.

[23] Iris Staging of CKD. http://www.iris-kidney.com/pdf/2_IRIS_Staging_of_CKD_2023.pdf.

[24] Diagnosing, Staging, and Treating Chronic Kidney Disease in Dogs and Cats. http://www.iris-kidney.com/pdf/IRIS_Pocket_Guide_to_CKD_2023.pdf.

[25] Griffin S. (2020). Feline abdominal ultrasonography: What’s normal? What’s abnormal? renal pelvis, ureters and urinary bladder. J. Feline Med. Surg..

[26] Haagmans B.L., Egberink H.F., Horzinek M.C. (1996). Apoptosis and t-cell depletion during feline infectious peritonitis. J. Virol..

[27] Takano T., Hohdatsu T., Hashida Y., Kaneko Y., Tanabe M., Koyama H. (2007). A “possible” involvement of TNF-alpha in apoptosis induction in peripheral blood lymphocytes of cats with feline infectious peritonitis. Vet. Microbiol..

[28] Dean G.A., Olivry T., Stanton C., Pedersen N.C. (2003). In vivo cytokine response to experimental feline infectious peritonitis virus infection. Vet. Microbiol..

[29] Cianci R., Massaro M.G., De Santis E., Totti B., Gasbarrini A., Gambassi G., Giambra V. (2023). Changes in lymphocyte subpopulations after remdesivir therapy for COVID-19: A brief report. Int. J. Mol. Sci..

[30] Yan Y., Li J., Jiao Z., Yang M., Li L., Wang G., Chen Y., Li M., Shen Z., Shi Y. (2023). Better therapeutic effect of oral administration of GS441524 compared with GC376. Vet. Microbiol..

[31] Zwicklbauer K., von la Roche D., Krentz D., Kolberg L., Alberer M., Hartmann K., von Both U., Härtle S. (2024). Characterization of the lymphocyte response in cats with feline infectious peritonitis during antiviral treatment using the smart tube technology for flow cytometry in feline full blood samples. J. Vet. Intern. Med..

[32] Zwicklbauer K., von la Roche D., Krentz D., Kolberg L., Alberer M., Zablotski Y., Hartmann K., von Both U., Härtle S. (2024). Adapting the smart tube technology for flow cytometry in feline full blood samples. Front. Vet. Sci..

[33] Buchta K., Zuzzi-Krebitz A., Zwicklbauer K., Bergmann M., Dorsch R., Hofmann-Lehmann R., Meli M.L., Spiri A.M., Matiasek K., Zablotski Y. (2025). Ein-Jahres-Follow-Up von Katzen nach Therapie der felinen infektiösen Peritonitis mit oralem GS-441524 für 42 versus 84 Tage. Tierärztliche Prax. Ausg. K Kleintiere/Heimtiere.

[34] Mateos González M., Sierra Gonzalo E., Casado Lopez I., Arnalich Fernández F., Beato Pérez J.L., Monge Monge D., Vargas Núñez J.A., García Fenoll R., Suárez Fernández C., Freire Castro S.J. (2021). The prognostic value of eosinophil recovery in COVID-19: A multicentre, retrospective cohort study on patients hospitalised in spanish hospitals. J. Clin. Med..

[35] Zimmermann N., Rothenberg M.E. (2015). Mechanism of enhanced eosinophil survival in inflammation. Blood.

[36] Ian M., Williams C. (2000). Drug-induced eosinophilia. Pharm. J..

[37] Tekes G., Thiel H.J. (2016). Feline coronaviruses: Pathogenesis of feline infectious peritonitis. Adv. Virus. Res..

[38] Cony F.G., Pereira V.C., Slaviero M., Lima R.P., de Castro L.T., de Moraes J.T.R., Aliardi J.M.G., Driemeier D., Sonne L., Panziera W. (2024). Anatomopathological characterization of hepatic lesions of feline infectious peritonitis in cats. J. Comp. Pathol..

[39] Malbon A.J., Fonfara S., Meli M.L., Hahn S., Egberink H., Kipar A. (2019). Feline infectious peritonitis as a systemic inflammatory disease: Contribution of liver and heart to the pathogenesis. Viruses.

[40] Xie J., Wang Z. (2021). Can remdesivir and its parent nucleoside gs-441524 be potential oral drugs? an in vitro and in vivo dmpk assessment. Acta Pharm. Sin. B.

[41] Zampino R., Mele F., Florio L.L., Bertolino L., Andini R., Galdo M., De Rosa R., Corcione A., Durante-Mangoni E. (2020). Liver injury in remdesivir-treated COVID-19 patients. Hepatol. Int..

[42] Kramer J.W., Hoffman W.E., Kaneko J.J., Harvey J.W., Bruss M.L. (1997). Clinical Enzymology.

[43] Levy J.K., Crawford P.C., Werner L.L. (2006). Effect of age on reference intervals of serum biochemical values in kittens. J. Am. Vet. Med. Assoc..

[44] Hall J.A., Yerramilli M., Obare E., Yerramilli M., Jewell D.E. (2014). Comparison of serum concentrations of symmetric dimethylarginine and creatinine as kidney function biomarkers in cats with chronic kidney disease. J. Vet. Intern. Med..

[45] Baral R.M., Freeman K.P., Flatland B. (2021). Comparison of serum and plasma SDMA measured with point-of-care and reference laboratory analysers: Implications for interpretation of SDMA in cats. J. Feline Med. Surg..

[46] Sargent H.J., Elliott J., Jepson R.E. (2021). The new age of renal biomarkers: Does sdma solve all of our problems?. J. Small Anim. Pract..

[47] Harley L., Langston C. (2012). Proteinuria in dogs and cats. Can. Vet. J..

[48] Furbish A., Allinder M., Austin G., Tynan B., Byrd E., Gomez I.P. (2024). Peterson. First analytical confirmation of drug-induced crystal nephropathy in felines caused by GS-441524, the active metabolite of remdesivir. J. Pharm. Biomed. Anal..

[49] Kent A.M., Guan S., Jacque N., Novicoff W., Evans S.J.M. (2024). Unlicensed antiviral products used for the at-home treatment of feline infectious peritonitis contain GS-441524 at significantly different amounts than advertised. J. Am. Vet. Med. Assoc..

[50] Friedel D.M., Cappell M.S. (2023). Diarrhea and coronavirus disease 2019 infection. Gastroenterol. Clin. N. Am..

[51] Wu Y., Cheng X., Jiang G., Tang H., Ming S., Tang L., Lu J., Guo C., Shan H., Huang X. (2021). Altered oral and gut microbiota and its association with SARS-CoV-2 viral load in COVID-19 patients during hospitalization. NPJ Biofilms Microbiomes.

